# Spatial-Memory Formation After Spaced Learning Involves ERKs1/2 Activation Through a Behavioral-Tagging Process

**DOI:** 10.1038/s41598-019-57007-4

**Published:** 2020-01-09

**Authors:** Ramiro Tintorelli, Pablo Budriesi, Maria Eugenia Villar, Paul Marchal, Pamela Lopes da Cunha, Julieta Correa, Martin Giurfa, Haydée Viola

**Affiliations:** 1 Laboratorio de Memoria, Instituto de Biología Celular y Neurociencia “Prof. E. De Robertis” (IBCN), Facultad de Medicina, UBA-CONICET, Buenos Aires, Argentina; 2 Institut de Génomique Fonctionnelle, CNRS, F-34094 Montpellier, cedex 05 France; 3 Centre de Recherches sur la Cognition Animale (CRCA), Centre de Biologie Intégrative (CBI), Université de Toulouse, CNRS, UPS, Toulouse, F-31062 cedex 09 France; 4 College of Bee Science, Fujian Agriculture and Forestry University, Fuzhou, 350002 China; 5 Departamento de Fisiología, Biología Molecular y Celular “Dr. Héctor Maldonado” (FBMC), Facultad de Ciencias Exactas y Naturales, UBA, Buenos Aires, Argentina

**Keywords:** Long-term memory, Spatial memory

## Abstract

The superiority of spaced over massed learning is an established fact in the formation of long-term memories (LTM). Here we addressed the cellular processes and the temporal demands of this phenomenon using a weak spatial object recognition (wSOR) training, which induces short-term memories (STM) but not LTM. We observed SOR-LTM promotion when two identical wSOR training sessions were spaced by an inter-trial interval (ITI) ranging from 15 min to 7 h, consistently with spaced training. The promoting effect was dependent on neural activity, protein synthesis and ERKs1/2 activity in the hippocampus. Based on the “behavioral tagging” hypothesis, which postulates that learning induces a neural tag that requires proteins to induce LTM formation, we propose that retraining will mainly retag the sites initially labeled by the prior training. Thus, when weak, consecutive training sessions are experienced within an appropriate spacing, the intracellular mechanisms triggered by each session would add, thereby reaching the threshold for protein synthesis required for memory consolidation. Our results suggest in addition that ERKs1/2 kinases play a dual role in SOR-LTM formation after spaced learning, both inducing protein synthesis and setting the SOR learning-tag. Overall, our findings bring new light to the mechanisms underlying the promoting effect of spaced trials on LTM formation.

## Introduction

Repeating a given experience does not always result in better memory of it. The time between experiences is crucial for the formation of a lasting memory. Since the pioneering work of Ebbinghaus^1^ to date, more than three hundred studies on verbal learning in humans have been performed leading to the conclusion that retention increases when the interval between learning sessions increases (see Cepeda *et al*.^2^). These and other observations led to the distinction between massed and spaced training, which rely on repeated short and long inter-trial intervals, respectively, and to the demonstration that the latter induces more robust memories than the former. This discovery was confirmed in various animal models as diverse as Aplysia, fly, bee, rodents and non-human primates trained in diverse learning protocols and contexts^3–9^. After a century of experimentation in this area, two major conclusions can be drawn: (1) the promnesic phenomenon induced by spaced training is evolutionarily conserved and, (2) the neurobiological bases of this phenomenon are not clearly known.

There are three well-known cognitive theories proposed to explain the superiority of spaced over massed training. While the first emphasizes the information coding processes, the second is based on the need to evoke the information learned at the time of the new training. The third considers that deficient processing of the information learned in massed learning would result in information loss^9^. Concerning this last theory, emerging data from behavioral and neuroscience studies point to memory consolidation as a potential process contributing to the advantages of spaced training. The classical theory of memory consolidation posits that the newly acquired information initially goes through a period of fragility and is stabilized with time, giving rise to long-term memory (LTM). During this period, different molecular and cellular changes occur in places where memory is formed, which affects that storage^10^.

A necessary condition for LTM formation is the induction of the synthesis of plasticity-related proteins (PRPs)^10–13^. This synthesis occurs when the acquired information contains a degree of novelty or stress, which activates attention systems^10,14^. However, weak learning experiences can utilize the proteins whose synthesis has been induced by other events adjacent in time to consolidate a memory trace. Synaptic plasticity and also learning and memory require input specificity for the encoding and storage of the information. Thus, in analogy to the synaptic tagging and capture hypothesis^15^, we postulated the behavioral tagging (BT) hypothesis^16^ proposing that a learning session sets a learning-tag within task-specific neurons, where plasticity proteins can be captured to establish LTM^17,18^. The processes involved in the formation or improvement of LTM by retraining are frequently studied using training protocols with multiple trials and/or sessions. However, it has been sometimes observed that repetitions do not always contribute to improve memory^6,19^. Here, we used the spatial object recognition (SOR) task, which requires that animals learn the spatial location of objects in an arena and react afterward to changes in location, showing thereby their spatial memory, and which is hippocampus-dependent^20^. We used two consecutive weak SOR training sessions (wSOR) and studied the mechanisms underlying the “lag effect”, *i.e*. the fact that longer intervals between sessions tend to produce better learning than shorter intervals (see Carpenter^21^). Based on the BT hypothesis, we suggest that retraining will mainly retag the sites initially labeled by the prior training. Moreover, we postulate that PRPs required for memory consolidation can be synthesized as a result of the sum or synergy of the consecutive weak training sessions when they are experienced within an appropriate temporal window. We thus aimed at determining if LTM promotion achieved by retraining relies on these two processes and if blocking any of them abolishes such a promotion. In addition, as the activation of extracellular regulated kinases 1/2 (ERKs1/2) or protein kinase A (PKA) after retraining is associated with an improvement of memory^6,8,22–26^, we studied the involvement of protein synthesis in the promotion of LTM by retraining, and the role of ERKs1/2 in either the tag setting or the protein synthesis process. We observed SOR-LTM promotion when two identical wSOR training sessions, which individually induce short-term memory (STM) but not LTM, were spaced by an inter-trial interval (ITI) ranging from 15 min to 7 h. The promoting effect was dependent on neural activity and protein synthesis. Moreover, our results suggest that ERKs1/2 activation in the dorsal hippocampus has a dual role, being a critical step for PRP synthesis and for the setting of the SOR learning-tag.

## Results

### Two consecutive weak SOR sessions induce LTM when spaced between 15 min and 7 h

**Figure 1:**
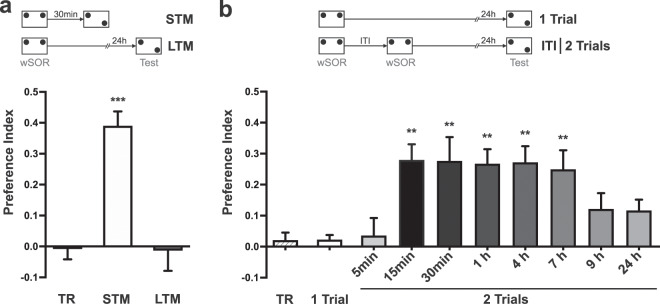
A single weak SOR training session induces SOR-STM but not SOR-LTM; retraining with a second SOR session within a critical time window ranging from 15 min to 7 h is effective to promote LTM. (**a**,**b**) show the preference index, expressed as mean ± SEM, registered in a training session (TR) or a test session performed 30 min or 24 h after training. (**a**) Rats were exposed to a 4-min wSOR training session (TR, n = 12) and independent groups were tested either 30 min (STM, n = 12) or 24 h (LTM, n = 12) after training. Newman-Keuls analysis after one-way ANOVA, F_(2,33)_ = 21.51; ***p < 0.001 vs. TR and LTM. (**b**) One-Trial group (n = 17) received a single 4-min wSOR training. Animals in the 2-Trials group were trained with two 4-min SOR sessions spaced by different ITIs spanning from 5 min to 24 h (5 min, n = 10; 15 min, n = 10; 30 min, n = 11; 1 h, n = 15; 4 h, n = 13; 7 h, n = 18; 9 h, n = 13; 24 h, n = 13). Representative first training session (TR, n = 18). Dunnett’s test after one-way ANOVA, F_(9,128)_ = 5.780; **p < 0.01 vs. 1 Trial and **p < 0.01 vs. TR.

We performed a wSOR training during which rats explored two identical objects inside an arena for 4 min. In the test session, one of the objects was displaced to a novel location in the same context, and we measured the exploration time allocated to both objects. Figure 1a shows that the group of rats trained with a single wSOR session and tested 30 min later explored more the object in the novel location, so that their preference index was significantly higher than that calculated for the training session (TR), exhibiting therefore SOR-STM. However, a parallel group of rats trained in the same wSOR task but tested 24 h later did not show SOR-LTM (p < 0.001 STM vs. other groups). In contrast, LTM formation was promoted when animals experienced a second and identical wSOR training session spaced by an inter-trial interval (ITI) ranging from 15 min to 7 h, (Fig. 1b; p < 0.01 vs. TR and 1 Trial). On the contrary, the same second wSOR session was ineffective to promote LTM if it was spaced from the first one by an ITI of 5 min, 9 h or 24 h (p > 0.05 vs. TR and 1 Trial), defining a critical time window in which the retraining protocol is effective for SOR-LTM formation.

### The promoting effect of retraining on SOR-LTM formation depends on neural activation and protein synthesis in the dorsal hippocampus

**Figure 2:**
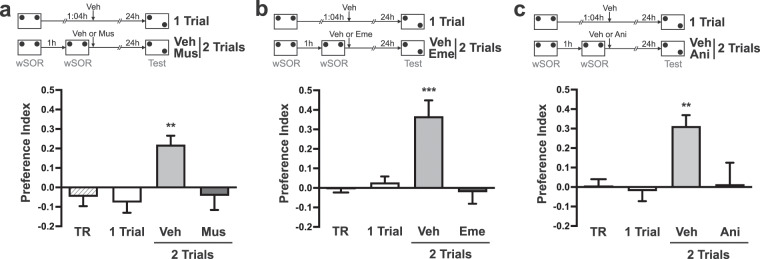
Inhibition of neural activation or protein synthesis in the dorsal hippocampus prevents SOR-LTM formation induced by retraining. (**a**–**c**), show the preference index as mean ± SEM in a first training session (TR), which is representative for all groups, and in a test session. (**a**) Independent animals were submitted to two identical wSOR training sessions spaced by 1 h and received bilateral infusions of either vehicle (2 Trials Veh, n = 10) or muscimol (2 Trials Mus, n = 7) in the dorsal hippocampus, immediately after the second training session. They were tested 24 h later. Animals exposed to a single wSOR training session (1 Trial, n = 8) received a vehicle infusion 65 min after that and were tested 24 h later. Training session (TR, n = 10). Newman–Keuls analysis after one-way ANOVA, F_(3,31)_ = 7.323; **p < 0.01 vs. all other groups. (**b**) One-Trial group injected with vehicle (n = 6) and 2-Trials group injected with vehicle (Veh, n = 8) or emetine (Eme, n = 6) immediately after the second training session were tested 24 h later. Training session (TR, n = 8). Newman–Keuls analysis after one-way ANOVA, F_(3,24)_ = 11.88; ***p < 0.001 vs. all other groups. (**c**) One-Trial group inje**c**ted with vehicle (n = 14) and 2-Trials group injected with vehicle (Veh, n = 14) or anisomycin (Ani, n = 10) immediately after the second training session were tested 24 h later. Training session (TR, n = 14). Newman–Keuls analysis after one-way ANOVA, F_(3,48) = _6.864; **p < 0.01 vs. all other groups.

In the previous experiment, we showed that rats trained with two wSOR sessions spaced by 1 h form a LTM observable 24 h after training. We then used this ITI and performed hippocampal infusions of vehicle, the neural blocker muscimol, or the protein-synthesis inhibitors emetine or anisomycin after the second training session to determine the effect of these inhibitions on SOR-LTM formation assessed 24 h after training. Figure 2a shows that rats infused with vehicle expressed SOR-LTM (p < 0.01 vs. TR) while rats infused with muscimol did not express it (p < 0.01 vs. Veh). Moreover, the promotion of SOR-LTM induced by retraining was blocked by the intra-hippocampal administration of emetine (p < 0.001 vs. Veh) and anisomycin (p < 0.01 vs. Veh) (Fig. 2b,c, respectively). These results indicate that the formation of SOR-LTM induced by retraining requires hippocampal activity and the induction of protein synthesis in the dorsal hippocampus.

### The learning-tag induced by wSOR is transient and depends on ERKs1/2 activation in the dorsal hippocampus

**Figure 3:**
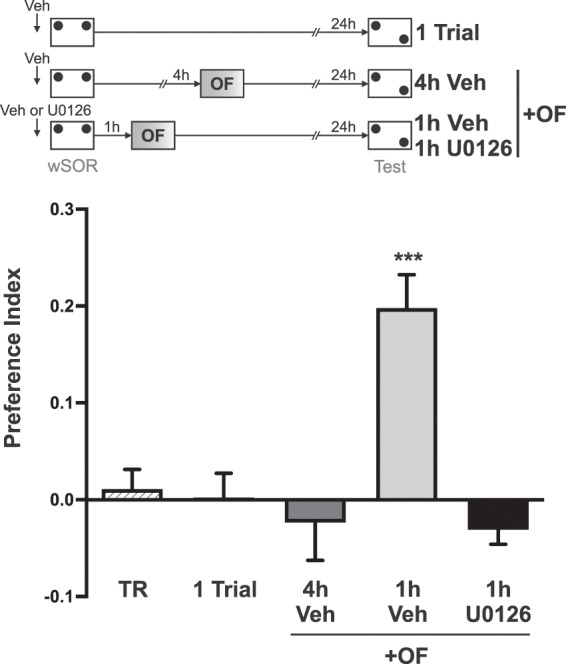
Exploration of an open field within a critical time window induces SOR-LTM formation and that is prevented by hippocampal inhibition of ERKs1/2. (Top) The flow chart shows the experimental protocol using wSOR and open field. One-Trial animals (n = 10) received bilateral dorsal hippocampus infusions of vehicle 15 min previous to wSOR training session and were tested the following day. Independent animals received bilateral dorsal hippocampus infusions of either vehicle or U0126 15 min before wSOR training session and were exposed to a novel OF session either 4 h (4 h Veh, n = 9) or 1 h (1 h Veh, n = 10; 1 h U0126, n = 12) after wSOR. Training session (TR, n = 11) is representative for all groups. Data are expressed as mean ± SEM. Newman–Keuls analysis after one-way ANOVA, F_(4,47) = _12.05; ***p < 0.001 for all other groups.

In a previous work, we coupled a wSOR training session, similar to the one used here, with an open-field (OF) session, and showed that the latter promotes the formation of SOR-LTM through the mechanism of behavioral tagging, which involves the setting of a learning tag by the wSOR training and the provision of the PRPs by OF exposure^27^. Thus, we used this protocol to show that this phenomenon occurs within a critical temporal window between the tasks and studied the molecular requirements of this process. We decided to explore the role of ERKs1/2 in establishing the SOR learning-tag because these kinases are required specifically for the setting of synaptic-tags associated with long-term depression (LTD)^28,29^, a cellular-plasticity model associated with the acquisition of spatial memory for object location in rodents^30,31^. We exposed rats to a 5 min OF session 1 h or 4 h after a wSOR training session. The group of rats exposed to OF 1 h after wSOR expressed SOR-LTM when they were tested 24 h after training (Fig. 3, p < 0.001 vs. TR). In contrast, control animals that were not exposed to the OF, and the group that was exposed to the novel OF 4 h after wSOR did not express SOR-LTM (Fig. 3, p < 0.001 vs. 1 h Veh). Moreover, the promoting effect of OF experienced 1 h after wSOR was prevented by the infusion of the specific MEK inhibitor U0126 15 min before wSOR training session (Fig. 3, p < 0.001 vs. 1 h U0126). This experiment suggests that the initial wSOR training session induces a learning tag that depends on ERKs1/2 activation. In addition, the results from rats infused with vehicle into the hippocampus indicate that the wSOR learning tag is no longer active 4 h after training, which is in agreement with our previous results obtained in non-cannulated rats^27^. Overall, these results indicate that during the ITI of 4 h separating two wSOR sessions, an additional process other than tag setting occurs given that LTM is formed under these conditions (Fig. 1b).

### The promoting effect of wSOR retraining on SOR-LTM formation depends on a dual role of ERKs1/2 activation in the dorsal hippocampus

**Figure 4:**
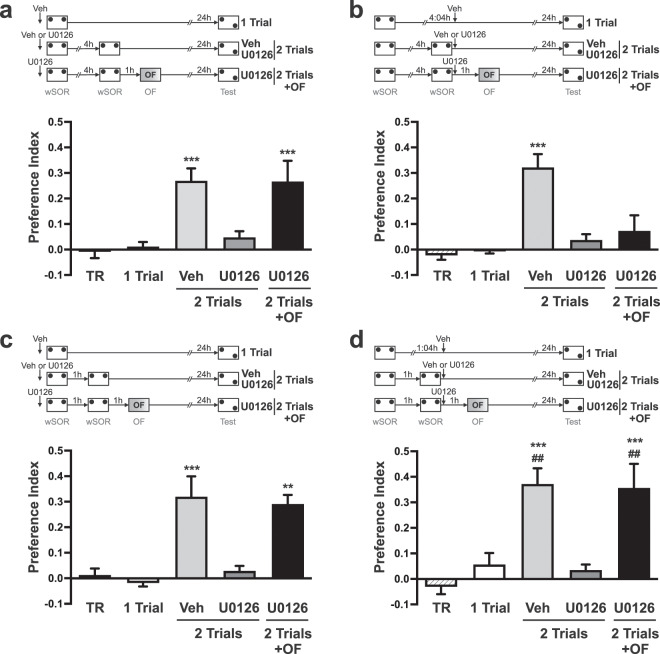
Hippocampal inhibition of ERKs1/2 prevents SOR-LTM formation induced by wSOR retraining, acting on learning-tag and protein synthesis processes. (**a**–**d**) show the preference index as mean ± SEM registered in the first training session (TR), which is representative for all groups, or in the test session performed 24 h after training. (**a**) One-Trial group (n = 8) was injected with vehicle 15 min before a single wSOR training session and tested on the following day. Independent animals received intra-dorsal hippocampus infusions of vehicle (n = 11) or U0126 (n = 9) 15 min before being subjected to two identical wSOR training sessions spaced by 4 h; another group was also exposed to an OF session 1 h after both training sessions (n = 6). Training session (TR, n = 12). SOR-LTM was tested 24 h after the second training session. Newman–Keuls analysis after one-way ANOVA, F_(4,41)_ = 12.18; ***p < 0.001 vs. TR, 1 Trial and 2 Trials U0126. (**b**) One-Trial group (n = 11) was injected with vehicle 4 h after a single wSOR training session and tested on the next day. Independent animals were subjected to two identical wSOR sessions spaced by 4 h and immediately after that, they received bilaterally infusions of either vehicle (n = 18) or U0126 (n = 15); another group was also exposed to an OF session 1 h after that training (n = 10). Training session (TR, n = 18). SOR-LTM was tested 24 h after the second training session. Newman–Keuls analysis after one-way ANOVA, F_(4,67)_ = 16.30; ***p < 0.001 vs. all other groups. (**c**) The experimental protocol is similar to (**a)**, except that the ITI is 1 h. One-Trial group of animals (n = 10), 2-Trials group of rats infused with vehicle (n = 13) or U0126 (n = 11) and the retrained group plus an OF session (n = 7). Training session (TR, n = 13). Newman–Keuls analysis after one-way ANOVA, F_(4,49)_ = 11.64; **p < 0.01; ***p < 0.001 vs. TR, 1 Trial and 2 Trials U0126. (**d**) The experimental protocol is similar to (**b**), except that the ITI is 1 h. One-Trial group of animals was injected with vehicle 1 h after training (n = 6), 2-Trials group of rats infused with vehicle (n = 11) or U0126 (n = 10) and the retrained group plus an OF session (n = 8). Training session (TR, n = 12). Newman–Keuls analysis after one-way ANOVA, F_(4,42)_ = 13.49; ***p < 0.001 vs. TR and 2 Trials U0126; ^##^p < 0.01 vs. 1 Trial.

We next assessed whether ERKs1/2 activation also participates in the regulation of protein synthesis required to form LTM after retraining. In order to ensure that the learning-tag induced by the first wSOR session has decayed at the moment of the second session, we trained rats with an ITI of 4 h. Note that this ITI resulted in LTM when rats were trained with two wSOR sessions (Fig. 1b and Veh group in Fig. 4a). Despite the fact that the second wSOR induced its learning-tag, the local infusion of U0126 15 min before the first wSOR session impaired the SOR-LTM (Fig. 4a, p < 0.001 vs. Veh), suggesting that ERKs1/2 was also involved in the process leading to the synthesis of PRPs. The inhibitory effect of U0126 was rescued by an OF session performed after the second wSOR session, which contributed PRPs to the second learning tag.

Infusion of U0126 in the dorsal hippocampus immediately after the second wSOR session impaired the SOR-LTM when an ITI of 4 h separated the two training sessions (Fig. 4b, p < 0.001 vs. Veh), consistently with an inhibition of the SOR learning-tag by this drug. This effect was not rescued by a novel OF session experienced 1 h after retraining (Fig. 4b, p < 0.001 vs. Veh). These results suggest that the intact learning-tag induced by the second wSOR session, which was spaced by 4 h from the first one, was necessary to utilize the PRPs provided by the novel experience.

When the ITI between wSOR sessions was 1 h, and thus sufficient for the first learning-tag to persist until the second session and to promote LTM (see Fig. 1b, and Veh group in Fig. 4c), the infusion of U0126 either before the first (Fig. 4c) or immediately after the second session (Fig. 4d) impaired SOR-LTM formation (p < 0.001 U0126 vs. Veh). In both cases, the exposure to an OF 1 h after retraining rescued the SOR-LTM. In the first case (U0126 infusion before the first wSOR session), LTM rescue was due to the provision of PRPs contributed by the OF session to the tag induced by the second wSOR session (Fig. 4c, p < 0.01 vs. 2 Trials U0126). This assumption was explicitly tested by administering emetine after the OF session. Inhibition of protein synthesis by emetine caused an amnesic effect, which was not present in control animals that experienced a vehicle injection after the OF session (see Supplementary Fig. S1). This result thus confirmed that the OF session contributed the PRPs necessary to rescue SOR-LTM. In the second case (U0126 infusion after the second wSOR session), LTM rescue can be explained by the supply of PRPs induced by the OF session to the learning-tag set by the first wSOR session, which was still available (Fig. 4d, p < 0.001 vs 2 Trials U0126). These results suggest that with an ITI of 1 h, the injection of U0126 either before the first or after the second wSOR session prevented PRP synthesis so that no SOR-LTM formation was observed. The inhibition of tag setting by U0126 was therefore overcome in our retraining protocol because at least one tag was always preserved and available for the PRPs induced by OF exposure.

## Discussion

In this work, we described the temporal window of efficacy for the promotion of SOR-LTM after a retraining protocol using two consecutive weak training sessions. This promoting effect depends on hippocampal activity and protein synthesis and requires, in addition, the activation of ERKs1/2 at the time of the first and the second wSOR session. Our results suggest that ERKs1/2 activity is probably needed to induce the protein synthesis necessary to consolidate SOR-LTM. In addition, ERKs1/2 activity is also an essential step for the setting/maintenance of the SOR-learning tag. Based on these results, we postulated that a process of behavioral tagging (BT) operates in the formation of SOR-LTM after retraining, and that ERKs1/2 activity plays a dual role in it, acting both at the level of tag setting and maintenance and PRP synthesis.

**Figure 5:**
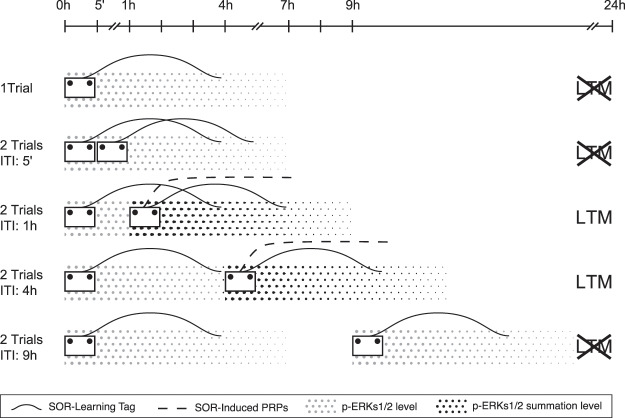
A model of the effect of different retraining protocols on SOR-LTM formation. The model is proposed on the basis of the present findings and the BT hypothesis, which postulates the requirement of the temporal and spatial convergence of a learning tag (solid line) set by the training, and synthesis of plasticity-related proteins (PRPs; dashed line), in order to promote the formation of LTM 24 h later. Such a convergence is indicated by the intersection of the PRPs and the tag lines, in which case SOR-LTM is observed. For PRP synthesis to occur, we suggest that activated ERKs1/2 levels (p-ERKs1/2, dotted area) induced by each weak SOR (wSOR) training sessions and lasting ca. 7 h should be subjected to an additive or synergistic process (bolder dot area) if separated by an appropriate inter-trial interval (ITI) ranging from 15 min to 7 h. The timeline (top: 0–24 h) indicates the time points of the experimental procedures. The rows show different cases varying from a single wSOR session (upper row) to two wSOR sessions separated by different ITIs (5 min - 9 h). The first row shows that a single wSOR session does not induce LTM on the following day despite inducing tag setting and enhancing ERKs1/2 level as it would be insufficient to trigger the required synthesis of PRPs. Two consecutive and identical wSOR sessions separated by 5 min (second row) or 9 h (fifth row) do not induce LTM as no PRP synthesis would occur in either case. Although each session would tag the same cellular substrates, in the first case, levels of ERKs1/2 induced by the first session would not be further enhanced or extended by the second one to reach the threshold necessary for PRP synthesis. This would be due to the necessity of a minimal ITI for the machinery inducing ERKs1/2 activation by the second session to be operational. In the second case, the tag and ERKs1/2 levels of the first session decay over time during the long ITI and do not reach the second session, thus impeding protein synthesis. The third and fourth rows correspond to ITIs of 1 and 4 h, respectively, in which PRP synthesis would occur. In both cases, ERKs1/2 levels would be enhanced and the same cellular substrates would be retagged by the second session. This convergence would lead to PRP synthesis necessary for LTM formation. However, as ERKs1/2 activity was not quantified under these circumstances, alternative explanations (besides addition or synergy in ERKs1/2 activation level) to explain how ERKs1/2 functionality may relate to wSOR training and PRP synthesis cannot be excluded.

Our results show that rats trained with a single wSOR session do not form SOR-LTM; however, when they were exposed to two identical wSOR sessions separated by an ITI ranging between 15 min to 7 h, they exhibited SOR-LTM. A critical step in the establishment of durable memories is the synthesis of proteins. In accordance with this, we observed that the infusion of the protein synthesis inhibitors anisomycin and emetine in the dorsal hippocampus, immediately after the second wSOR training session, fully blocked the expression of LTM. A similar result was observed after infusing muscimol, a GABA_A_ receptor agonist that temporarily silences the infused area. Because the same behavioral output was observed after preventing the activation of ERKs1/2 through U0126 infusion in the hippocampus, we suggest that these kinases enable the process of protein synthesis after retraining. In that sense, the summation of the biochemical effects induced by retraining would be necessary to promote LTM. If this were the case, the observed ineffectiveness of the short 5 min ITI to promote SOR-LTM could be due to an incapacity of the second training session to enhance and/or extend the levels of ERKs1/2 activation that would be required for memory consolidation^9^. This molecular explanation constitutes an alternative view to the hypothesis suggesting that memories established on consecutive trials interfere with each other’s during shorter ITIs corresponding to a window of high susceptibility to interference^32^. To further discriminate between these two points of view, we observed that OF promoted SOR-LTM when it was experienced one hour after two wSOR sessions spaced by 5 min (see Supplementary Fig. S2). This result suggests that a short ITI does not interfere with a fundamental process that could not be overcome by providing PRPs, such as the tag setting process; in contrast, it seems to impair mechanisms associated with the synthesis of PRPs required for memory consolidation. In the scheme proposed to account for our findings (see Fig. 5), the absence of LTM after an ITI of 5 min does not result from interference between consecutive trials but from an absence of sustained or enhanced activity of ERKs1/2 induced by this ITI. ITIs higher than 7 h are also ineffective to promote LTM because the effects of the first training session would no longer persist until retraining. However, we do not discard the possibility that other processes triggered by the first and the second waves of ERKs1/2 activation (and not necessarily its sustained level) facilitate the triggering of PRP synthesis. In that sense, recent findings reported that repeated experiences in contextual fear conditioning or Morris water maze may be integrated within a time window of 5 h to possibly promote their LTM. Moreover, this depended on network activity and c-Fos expression, which was sufficient and necessary to determine what mice learn^33^.

An important issue in retraining protocols is to know whether the population of cells activated by the first training coincides with that activated by the second one. The use of fluorescent *in-situ* hybridization and confocal microscopy to monitor the subcellular distribution of the immediate-early gene Arc revealed that rats exposed sequentially to the same environment exhibited duplicated proportion of CA1 neurons with overlapping Arc expression with respect to animals exposed sequentially to two different environments^34,35^. Moreover, Attardo *et al*.^36^ used a fluorescent reporter of neural plasticity to image long-term cellular ensemble dynamics of live mice, and they observed that CA1 cell patterns representing the enriched environment were progressively stabilized after repeated episodes. As expected, exposure to the same environment evoked patterns about twice as high as those evoked by different environments. Also, Abdou *et al*.^37^ showed that assemblies in the basolateral amygdala and the auditory cortex overlap more if the associative fear experience is the same. On the other hand, if the task implies the association of context with different tones, the overlapping degree of the cellular assemblies was lower than that corresponding to the repetition of the same experience. Moreover, the authors suggested that engram-specific synaptic plasticity is crucial and sufficient for information storage and keeps the identity of the distinct overlapping memories. Thus, they showed that it was possible to erase a fear memory from an engram network without affecting other memories stored in the shared ensemble by resetting the plasticity in a synapse-specific manner.

The aforementioned findings highlight two main facts: the repetition of a given episode activates similar neural substrates as those used by the original one, and a given memory requires synaptic plasticity specificity. In line with this statement, the BT hypothesis offers a conceptual framework to explain how PRPs could be used at sites activated by training. In this framework, the formation of LTM relies on two key cellular processes: the synthesis of PRPs and the setting of a learning-tag^18,38^, which provides the specificity for the memory storage. It has been proposed that tag setting does not require protein synthesis, but is based on post-translational changes and re-assembly of the cytoskeleton that lead to changes in spine morphology^39^. Kinase activity was postulated as a necessary step in the tag setting after synaptic plasticity or learning experiences^18^.

The participation of the BT process in a SOR paradigm was already shown by Ballarini *et al*.^27^, who demonstrated that a single wSOR session could result in a SOR-LTM if associated with a novel OF exposure occurring to two hours after the training session. This phenomenon was dependent on the protein synthesis induced by the OF novelty. In the present work, we used this finding to assess the duration of the learning tag induced by the initial wSOR training session and to determine its dependence on ERKs1/2 activation (see Fig. 3). We observed that the infusion of the ERKs1/2 inhibitor U0126 in the dorsal hippocampus before the wSOR session impaired the SOR-LTM promotion induced by the OF exposure 1 h after the training session. This result suggests that ERKs1/2 activation at the moment of learning is necessary for the setting of the tag by the SOR session. We also confirmed in cannulated rats infused with the vehicle before the wSOR training session that OF exposure promoted SOR-LTM when given 1 h, but not 4 h, after wSOR session. Overall, our results suggest that the SOR learning-tag persists less than 4 h and depends, at least in part, on ERKs1/2 activity in the dorsal hippocampus.

Finally, we studied if ERKs1/2 activation is also involved in the processes of tag setting and induction of protein synthesis after retraining. As the second training session will mainly retag the sites labeled by the first session, and to further test if SOR-LTM formation after retraining needs an active learning-tag, we used a 4 h ITI protocol to ensure that the transient learning-tag induced by the first wSOR had declined. We observed that the local infusion of U0126 after the second wSOR session impaired the SOR-LTM, and also prevented memory promotion induced by a subsequent OF exposure. This result suggests that ERKs1/2 activity is required to set the SOR learning-tag and that in its absence, the PRPs induced by the OF exposure are ineffective for SOR-LTM formation. In contrast, when a 1 h ITI retraining protocol was used, the local infusion of U0126 after the second wSOR session did not impaired the OF promoting effect on SOR-LTM formation, because the PRPs induced by OF exposure could be used by the learning-tag set by the first wSOR session, which was still active during the OF session. On the other hand, the role of ERKs1/2 activity for signaling protein synthesis could be evidenced when U0126 was infused in the hippocampus before the first wSOR training session both with ITIs of 1 h and 4 h. In both cases, the inactivation of ERKs1/2 impaired SOR-LTM suggesting that even when the second learning-tag was active, because it was not reached by U0126, memory was not formed probably due to lack of protein synthesis. We speculate that PRPs required for memory consolidation can be synthesized as a result of the sum or synergy of two wSOR sessions that are experienced in an appropriate temporal window, and that ERKs1/2 activity is crucial for this phenomenon. This dynamic in protein synthesis is also compatible with a metaplasticity-like mechanism by which prior experience impacts subsequent learning^40^.

An important issue, both in synaptic plasticity models and in BT protocols, is the identification of the molecules responsible for setting the tags. Our results suggest that ERKs1/2 activation is required to set the SOR learning tag. These results are in accordance with the fact that ERKs1/2 are required specifically for the setting of synaptic-tags associated with LTD^28,29^, a cellular plasticity model associated with the acquisition of spatial memory for object location in rodents^30,31^. Also, hippocampal ERKs1/2, but not PKA, may serve as behavioral tags to promote LTM extinction of an aversive memory task^41^. In contrast, Moncada *et al*.^42^ showed that 𝛼CAMKII, PKA, and PKM𝜁, but not ERKs1/2, activities play an essential role in the setting of the learning tag resulting from an inhibitory avoidance task. This is in agreement with results showing the same kinase dependency of the synaptic tag induced by LTP protocols^28,29,43^, a cellular plasticity model associated with an inhibitory avoidance task^44^.

The involvement of ERKs1/2 in the formation of LTM after retraining found in our work is in agreement with other studies. In *Aplysia*, a 45-min interval between stimuli was effective for the induction of LTM for sensitization of the tail-elicited siphon withdrawal reflex, and for ERKs1/2 activation in the tail sensory neurons^8,19^. In olfactory conditioning of *Drosophila*, consisting of pairing odor with an electric shock, Pagani *et al*.^6^ demonstrated that the cycle of ERKs1/2 activation must decay to permit a resetting with the subsequent trial. Recently, Miyashita *et al*.^22^ demonstrated that this ERKs activation is required for the increased expression of c-fos and dCREB2 during spaced training. Moreover, Li *et al*.^45^ suggested that translocation of ERKs1/2 to the nucleus of mushroom body neurons is required for the consolidation of this LTM after retraining. ERKs1/2 activity is also a key step in LTM induced by retraining in rodents. The infusion of a MEK blocker into the striatum, both at the time of the second training and 3 h later, impaired the enhancement of an inhibitory-avoidance memory induced by retraining^46^. However, Parsons and Davis^23^ reported the activation of ERKs1/2 in the amygdala one hour after the first fear-training session but not after the second one. Using a recognition-memory paradigm, similar to that used in the present work, Seese *et al*.^24^ observed that synaptic ERKs1/2 activation was associated with the formation of object-location memory after spaced training in mice, which are model for the fragile X syndrome.

In conclusion, we report the existence of a temporal window ranging from 15 min to 7 h between two wSOR sessions, which is effective to promote SOR-LTM. Our results suggest that ERKs1/2 activity is: (1) necessary to induce protein synthesis required for memory formation after retraining and, (2) relevant to set the SOR learning-tag, which marks specific sites activated by re-learning. Finally, and in addition to a great body of evidence showing that the BT process accounts for LTM promotion by novel or stressful experiences^18,38,47^, the present results highlight that the formation of LTM after wSOR retraining is also in line with the assumptions of the BT hypothesis (Fig. 5).

## Materials and Methods

### Subjects

Male adult Wistar rats between 2 and 3 months of age (weight, 200–350 g) obtained from the breeding colony maintained at the Faculty of Exacts and Natural Sciences of the University of Buenos Aires were used in this study. Animals were housed in groups of three per cage, with food and water available *ad libitum*, under a 12 h light/dark cycle (lights on at 07:00 A.M.) at a constant temperature of 23 °C. All behavioral testing was conducted during the light phase of the cycle. Animals were handled for 2 min for two consecutive days before each experiment to avoid emotional stress. During behavioral procedures, animals were individually moved from their home cages to the arena and returned immediately after each trial session. All experiments were conducted in accordance with the National Institutes of Health Guides for Care and Use of Laboratory Animals (Publication No. 80–23, revised 1996) and were approved by the Animal Care and Use Committee of the University of Buenos Aires (CICUAL).

### Drugs

All drugs supplied were purchased from Sigma (St. Louis, MO). The GABA_A_ agonist muscimol was applied to temporarily inactivate the dorsal hippocampus (0.1 µg of muscimol in 0.5 µl saline solution per side). The protein synthesis inhibitors used were anisomycin (80 µg of anisomycin, dissolved in HCl, diluted in saline, adjusted to pH 7.4 with NaOH, and infused in a volume of 0,8 µl per side) and emetine (50 µg in 1 µl saline solution per side). U0126 (0.4 μg diluted in 10% DMSO in saline and infused in a volume of 0,8 µl per side) was used as an ERKs1/2 inhibitor given that it blocks the kinase activity of MEK1/2, thus preventing the activation of MAP kinases p42 and p44 encoded by the erk2 and erk1 genes, respectively.

### Surgery and drug infusion

For cannulae implantation, rats were deeply anesthetized (70 mg/Kg ketamine and 7 mg/Kg xylazine). 22-G cannulae were stereotaxically aimed at the CA1 region of the dorsal hippocampus at coordinates A: −3.9 mm; L: ±3.0 mm; D: −3.0 mm, from Bregma^48^ (see Supplementary Fig. S3) and were cemented to the skull with dental acrylic. Animals received a subdermal application of analgesics and antibiotics during surgery (Meloxicam 0.2 mg/Kg, gentamicin 3 mg/Kg) and were allowed to recover from surgery for at least four days. Drugs were infused using a 30-G needle with its tip protruding 1.0 mm beyond the guide. The infusions needles were linked by an acrylic tube to a Hamilton microsyringe and the entire bilateral infusion procedure lasted about 2 min. Needles were left in place for one additional minute after infusion to minimize back-flow. Histological examination of cannulae placements was performed after the end of the behavioral procedures by the infusion of 0.5 µl of 4% methylene blue in saline solution. Animals were killed by decapitation 15 min after the infusion and their brains were sliced to verify the infusion area^49^. Only data from animals with correct cannulae implants (95%) were included in statistical analyses.

### Spatial object recognition (SOR) task

In the SOR task, animals familiarized with two objects in a specific spatial environment should recognize that one of them has changed its location with respect to its original position and the other object. Rats spend more time exploring the spatially displaced familiar object relative to a stationary familiar object, suggesting that they remember the location in which particular objects were previously encountered^50^.

The SOR arena was a 60 cm wide x 40 cm long x 50 cm high acrylic box, with different visual clues in its lateral white walls. The floor was white, the front wall was transparent and the back wall was hatched. For habituation to the context, all subjects explored the arena without objects for a 20 min daily session during two consecutive days before the training day. In the wSOR training session, two identical plastic or glass objects were included in the arena in two adjacent corners and animals were left to explore it for 4 min. In the test session, one of the objects was moved to a new position and animals were allowed to explore this context for 2 min. Exploration time for each object, defined as sniffing or touching it with the nose or forepaws, was measured using a hand stopwatch. Rats were excluded from the analysis when they explored one object more than 65% of the total object-exploration time during training sessions or when they did not reach 10 s in the total object-exploration time during the 2-min test session. Results are expressed as a preference index: [Exploration time of the object in a new location (Tn) - Exploration time of the object in the familiar location (Tf)] / [Tn + Tf]. Also, we calculated a preference index for the first training session (TR), considering Tf as the exploration time of the object that will be congruent in the test session and Tn the exploration time of the other object. In all cases, the preference index calculated for TR was not different from zero (p > 0.05), thus showing an initial absence of preference for exploring a particular location. A positive preference index in the test session, differing significantly that calculated for the TR, indicates the presence of memory. A representative mean ± SEM of the total object-exploration time during the first wSOR training session was 53.68 ± 1.87 s. It was 45.83 ± 1.67 s during the wSOR retraining session and 23.01 ± 0.77 s during the test session.

### Open field (OF) task

The OF task consists in placing an animal within an arena to record its locomotor and exploratory behavior in this novel spatial context. The arena was a 50 cm wide x 50 cm long x 39 cm high square box, with black plywood walls and floor divided into nine squares by white lines. The number of line crossings and rearings was measured in blocks of 1 min during 5 min under normal room lighting^16^.

### Data analysis

Behavioral data were analyzed by means of Newman-Keuls or Dunnett *post-hoc* comparison tests after one-way ANOVA. Analyses were performed in GraphPad Prism® version 8.00 (GraphPad Software, La Jolla, CA, USA). Effects were considered significant when p < 0.05. Results are presented as mean ± SEM.

## Supplementary information

Supplementary information.

## Data Availability

The data that support the findings of this study are available from the corresponding author upon reasonable request.
