# Supplementary material for: Spatial-Memory Formation After Spaced Learning Involves ERKs1/2 Activation Through a Behavioral-Tagging Process
Source: Sci Rep. 2020 Jan 9;10:98. doi: 10.1038/s41598-019-57007-4 (PMC6952433; doi:10.1038/s41598-019-57007-4)
Supplement: Supplementary file 1 — Supplementary information. [file 41598_2019_57007_MOESM1_ESM.pdf]

# Spatial-Memory Formation After Spaced Learning Involves ERKs1/2 Activation Through a Behavioral-Tagging Process.

Ramiro Tintorelli[1], Pablo Budriesi[1], Maria Eugenia Villar[1,2], Paul Marchal[3], Pamela Lopes da Cunha[1], Julieta Correa[1], Martin Giurfa[3,4] and Haydée Viola[1,5]

[1]Laboratorio de Memoria, Instituto de Biología Celular y Neurociencia "Prof. E. De Robertis" (IBCN), Facultad de Medicina, UBA-CONICET, Buenos Aires, Argentina; [2]Institut de Génomique Fonctionnelle, CNRS, F-34094 Montpellier cedex 05, France; [3]Centre de Recherches sur la Cognition Animale (CRCA), Centre de Biologie Intégrative (CBI), Université de Toulouse; CNRS, UPS, France; [4]College of Bee Science, Fujian Agriculture and Forestry University, Fuzhou 350002, China; and [5]Departamento de Fisiología, Biología Molecular y Celular "Dr. Héctor Maldonado" (FBMC), Facultad de Ciencias Exactas y Naturales, UBA, Buenos Aires, Argentina.

Correspondence and requests for materials should be addressed to H.V. (email: hviola@fmed.uba.ar)

## SUPPLEMENTARY INFORMATION

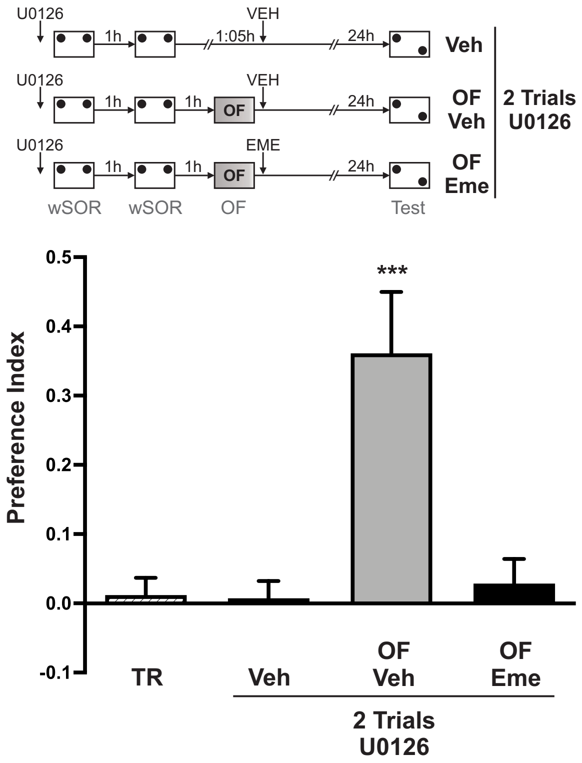


**Supplementary Figure S1. The rescuing effect of SOR-LTM by OF exposure under U0126 treatment depends on hippocampal protein synthesis.**
(Top) The flow chart shows the experimental protocol that consisted in training animals with two consecutive wSOR sessions spaced by 1h and testing them 24 h later for assessing the presence of SOR-LTM. All animals received bilateral dorsal hippocampus infusions of U0126 15 min before the first wSOR session. Three independent groups of animals were run in parallel. One group (n = 8) received an injection of vehicle 1:05 h after the wSOR retraining. The two other groups received a novel OF session 1 h after the wSOR retraining. Immediately after OF exposure, animals

in these two groups received bilateral hippocampal infusions of either vehicle (n=8) or emetine (n=7). Only the vehicle-injected animals showed SOR LTM, thus showing that PRPs contributed by the novel OF session were required for establishing this memory. Representative training session (TR, n=10). Data are expressed as mean ± SEM. Newman–Keuls analysis after one-way ANOVA, $F_{(3,29)} = 12.08$; ***p < 0.001 vs. all the other groups.

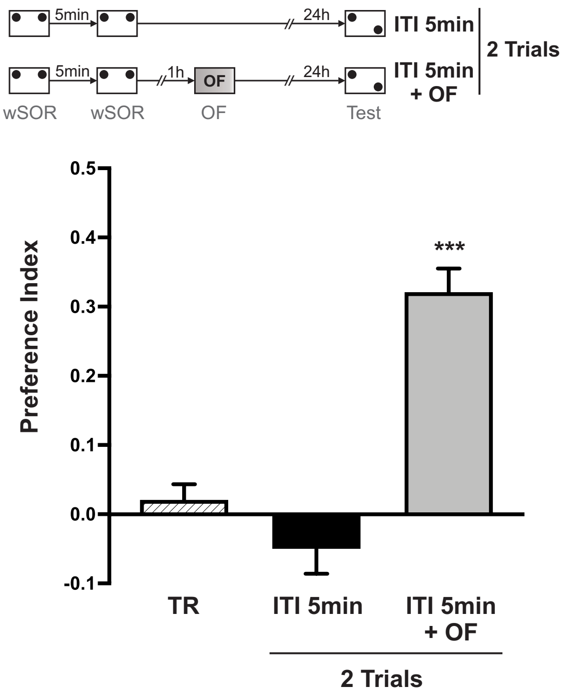


**Supplementary Figure S2. Exposure to an OF after two wSOR sessions spaced by a short interval (5 min) induces SOR-LTM formation.** (Top) The flow chart shows the experimental protocol that consisted in training two groups of animals with two consecutive wSOR sessions spaced by 5 min. One group (n = 8) did not experience an OF session while the other group (n = 10) experienced it one hour later. The latter, but not the former, exhibited SOR LTM 24 h later. Training session (TR, n=10). Data are expressed as mean ± SEM. Newman–Keuls analysis after one-way ANOVA, $F_{(2,25)} = 40.76$; ***p < 0.001 vs. all the other groups.

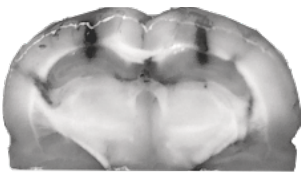

**Supplementary Figure S3. Representative picture showing the infusion area in the dorsal hippocampus.** Histological examination of cannulae placements was performed after the end of the behavioral procedures by the infusion of 0.5 µl of 4% methylene blue in saline solution.
